# Early flora colonization affects intestinal immunoglobulin G uptake in piglets, which may be mediated by NF-κB-FcRn pathway

**DOI:** 10.3389/fmicb.2023.1136513

**Published:** 2023-02-14

**Authors:** Fang Peng, Haihan Zhang, Xi He, Zehe Song

**Affiliations:** College of Animal Science and Technology, Hunan Agricultural University, Changsha, China

**Keywords:** newborn piglets, IgG transport, neonatal Fc receptor, NF-κB signaling pathway, flora colonization

## Abstract

**Introduction:**

The passive immunity of newborn piglets is mainly derived from immunoglobulin G (IgG) in breast milk, and the incomplete transfer of passive immune is considered to be an important cause of piglet death. This study was conducted to investigate the effect of early intestinal flora colonization on IgG uptake and its possible mechanism.

**Methods:**

The newborn piglets and IPEC-J2 cells were used to investigate the possible factors and regulatory mechanisms affecting intestinal IgG uptake. *In vivo*, all 40 piglets were euthanized on postnatal d 0, 1, 3, and 7, with 10 piglets per time. The blood sample, gastric contents, jejunal contents and mucosa were collected for analysis. *In vitro*, IPEC-J2 cells transwell culture system was used to establish the IgG transporter model to explore the specific regulatory mechanism of IgG transport.

**Results:**

Our results demonstrated that the intestinal IgG uptake was positively correlated with the expression of Neonatal Fc receptor (FcRn). With the increase of age, the intestinal flora of newborn piglets was gradually enriched. The function of intestinal genes also changes with the colonization of intestinal flora. We found that the expression trend of TLR2, TLR4 and NF-κB (P65) in intestine was consistent with that of FcRn. Furthermore, the *in vitro* results demonstrate that the NF-κB signaling pathway is involved in regulating FcRn-mediated IgG transmembrane transport.

**Discussion:**

Early flora colonization affects intestinal IgG uptake in piglets, which may be mediated by NF-κB-FcRn pathway.

## Introduction

In modern intensive production, piglets death accounted for more than 80% of pig mortality in whole production cycle, and caused huge economic losses to the industry. The reasons for high mortality rate in nursing period are highly sophisticated, among which the incomplete passive immune transfer is considered to be an important factor (Bandrick et al., 2014). The level of antibodies in newborn piglets’ body is basically zero, thus they are directly exposed to the risk of invasion by various pathogenic microorganisms after birth. However, the development of active immunity of newborn piglets is very slow, and their immune system does not protect until 7–8 days after birth. Thus, passive immunity must be obtained by sucking colostrum rich in antibodies to resist the invasion of various pathogenic bacteria (Kielland et al., 2015; Lauritsen et al., 2017).

Previous studies have shown that the passive immunity of piglets mainly comes from the immunoglobulin in breast milk, including immunoglobulin G (IgG), IgA, IgM and other types (Pierzynowska et al., 2020). The main immunoglobulin in colostrum within 3 days after delivery is IgG, reaching 60 mg/mL, accounting for 70–80% of the total immunoglobulin. IgG is the most important immune substance obtained from the mother of newborn piglets before the establishment of their own immune system, and it plays a very important role in the animal’s anti-infection immunity (Zeng et al., 2016; Bournazos and Ravetch, 2017; Mortensen et al., 2017). It is found that the serum IgG content of piglets after birth is positively correlated with the survival rate of piglets, and the serum IgG content of dead piglets is much lower than that of alive piglets (Czech et al., 2010). There is a strong correlation between the concentration of IgG in colostrum of sows and the mortality of piglets infected with intestinal and respiratory diseases. Obtaining sufficient maternal antibodies can significantly reduce the incidence and mortality of intestinal and respiratory diseases in piglets (Guo et al., 2016a,b; Andraud et al., 2018). Other studies have reported that the pre-weaning survival rate of suckling piglets can reach 91% when the serum IgG content is 22.52 mg/mL, while the weaning survival rate of suckling piglets is only 67% when the serum IgG content is equal to or less than 10 mg/mL (Cabrera et al., 2012). However, it is worth noting that the immunoglobulin in colostrum changed rapidly with the passing of postpartum time. It decreased by 30% within 3 h after delivery and nearly 90% after 24 h, which was mainly the decrease in IgG content. Therefore, it is necessary to obtain sufficient maternal IgG from colostrum quickly to ensure the survival and healthy growth of piglets.

Neonatal Fc receptor (FcRn) is the only receptor for specific IgG transport and plays a decisive role in IgG absorption (D'Hooghe et al., 2017). Some studies have reported that about 80% of IgG uptake in the small intestine is mediated by FcRn protein expressed on the surface of the small intestinal chorionic membrane (Kliwinski et al., 2013). Accumulating evidence demonstrated that NF-κB signaling pathway plays a pivotal role in the regulation of FcRn expression. Liu et al. (2007) reported that TNF-α improved FcRn expression and IgG transport efficiency across intestinal epithelial cells by activating NF-κB signaling pathway. Guo et al. (2016a,b) found five NF-κB (p65) binding sites by analyzing the sequence of porcine FcRn promoter region, and overexpression or activation of NF-κB signaling pathway by agonists significantly increased FcRn expression. It has been widely confirmed that TLR2 and TLR4 mediate the activation of NF-κB signaling pathway in immune and inflammatory responses. Moreover, studies have shown that the regulation of intestinal TLR2/4 in early life of newborn animals is mainly regulated by intestinal microbiota colonization (Round et al., 2011; Inoue et al., 2017). However, few studies have reported the effects of gut microbiota on passive immunity and IgG uptake in animals during early life.

Therefore, in this work, we analyzed the intestinal IgG transport and bacterial colonization of newborn piglets at different days of age, and constructed an *in vitro* IgG transport model using IPEC-J2 cells transwell culture system to analyze the specific regulatory mechanism of IgG transport in newborn piglets.

## Materials and methods

### Ethic approval and consent to participate

All procedures were approved by the Institutional Animal Care and Use Committee of Hunan Agricultural University.

### Animals experiment

**Figure 1:**
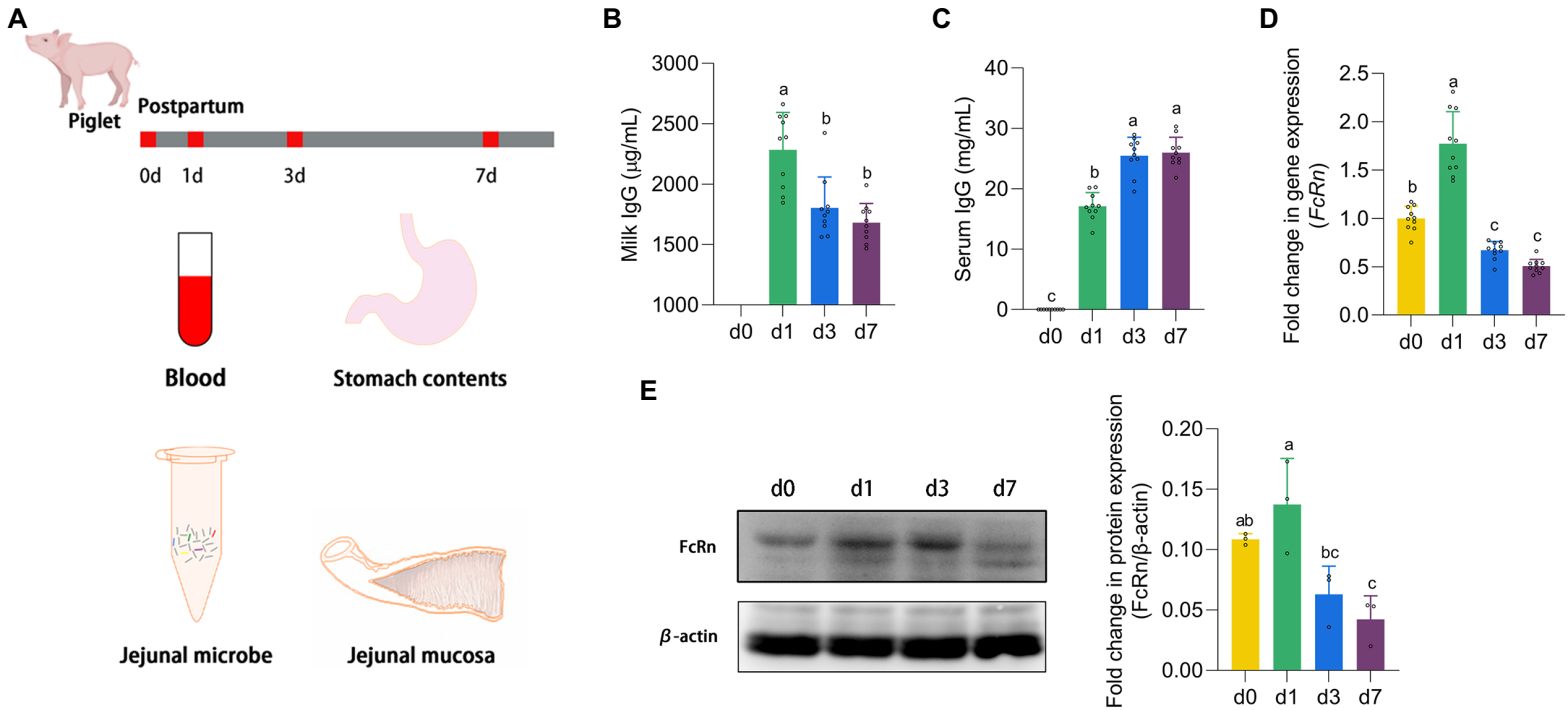
The expression of FcRn promoted the absorption of IgG. **(A)** Animal experiment design. **(B,C)** The IgG content in stomach milk and serum of piglets at four time points (d 0, 1, 3, and 7). **(D,E)** The mRNA and protein expression of FcRn in jejunal mucosa of piglets at four time points (d 0, 1, 3, and 7). Data were shown as means ± standard deviations. Mean value without the common letter on data bar in each figure indicated that the difference was significant (*p* < 0.05).

Newborn piglets (Landrace × Duroc × Yorkshire) were obtained from 4 sows (10 piglets per litter) with the similar parity (3 or 4 parities). The piglets were housed with their own mothers. All 40 piglets were euthanized on postnatal d 0, 1, 3, and 7, with 10 piglets per slaughter. All 30 piglets killed at d 1, 3, and 7 received breast milk except for 10 piglets at d 0. The blood sample was taken from the carotid artery and centrifuged at 3,000 rpm for 15 min at 4°C to obtain serum. Gastric contents (IgG in breast milk), jejunal contents and mucosa were collected and transferred into sterile precooled tubes, and then stored at −80°C until further analysis (Figure 1A).

### Cell culture

IPEC-J2 cells were obtained from the Type Culture Collection of the Chinese Academy of Sciences (Shanghai, China). The cells were cultured in Dulbecco’s modified Eagle medium (DMEM) supplemented with 10% fetal bovine serum (FBS) (HyClone, Utah, United States) and 1% Penicillin–Streptomycin (Beyotime Institute of Biotechnology, Shanghai, China). Cells were maintained at 37°C in a humidified incubator under 5% CO_2_.

### Establishment of *in vitro* transcellular model

IPEC-J2 cells were seeded in 96-well culture plates and grown to approximately 80% confluence. The cells were incubated with lipopolysaccharide (LPS) (0, 0.1, 0.5, 1, 5, 10 μg/mL) and lipoteichoic acid (LTA) (0, 1, 2.5, 5, 10, 20 μg/mL) in serum-free DMEM for 24 h, respectively, and their viabilities were determined by cell counting kit-8 assay. The maximum concentration that had no effect on cell viability was chosen as the final concentration of LPS and LTA for the following experiments.

IPEC-J2 cells were grown in 12-well transwell system and the changes of trans-epithelial electrical resistance (TEER) were determined using an epithelial voltohmmeter ERS-2 (Merck Millipore, United States). When the epithelial electrical resistance was measured until similar values were recorded on three consecutive measurements, the cells were incubated with LPS and LTA for 24 h, repectively. Then the electrical resistance of each treatment was measured.

Repeating the above procedure until the epithelial electrical resistance was stable. Then, porcine IgG (400 μg/mL) mixed with LPS or LTA or both not was added to the upper chamber of the transwell for 24 h at 37°C. Meanwhile, the opposite chamber was incubated in DMEM serum-free medium. Supernatants from the lower chamber of the transwell were collected at 1, 2, 4, 6, 12, 24 h after incubation. Meanwhile, cells were collected during these time periods.

### NF-κB inhibition assay

IPEC-J2 cells were grown at 70–80% confluence treated with or without 5 μM NF-κB inhibitor BAY11-7028 (Sigma-Aldrich, United States) for 1 h and then the cells were stimulated by LPS or LTA or both not for 2 h. Finally, cells were harvested for use.

### Enzyme-linked immunosorbent assay

The IgG levels in serum, gastric contents and cell culture medium supernatants, LPS and LTA levels in jejunal contents were determined using ELISA kits (Enzyme-linked Biotechnology, Shanghai, China) according to the manufacturer’s instructions.

### Absolute quantitative PCR

The total DNA from the jejunal content samples was extracted using QIAamp DNA Stool Mini Kit (Qiagen, Germany) following the manufacturer’s instructions. The PCR primer sequences were 338F: 5′-ACTCCTACGGGAGGCAGCA-3′ and 806R: 5′-GGACTACHVGGGTWTCTAAT-3′. The construction of plasmid standard and standard curve drawing were carried out by Paisonol Biotechnology Co., Ltd., Shanghai, China. Gene expression was determined on a real-time quantitative PCR system, using SYBR Green Premix Pro Taq HS qPCR kit (Accurate Biotechnology, Hunan, China). The formula for calculating the copy number is Ct = −KlogX+b. X represents copy number, K represents slope of standard curve, and b represents intercept of standard curve.

### Quantitative PCR

Total RNA was extracted from jejunal mucosa and IPEC-J2 cells using RNA rapid extraction kit and transcribed into cDNA using the Evo M-MLV RT Premix kit (Accurate Biotechnology, Hunan, China). The PCR primer sequences utilized for the determination of the gene expression were shown in Table 1. Gene expression was determined on a real-time quantitative PCR system, using SYBR Green Premix Pro Taq HS qPCR kit (Accurate Biotechnology, Hunan, China). Relative quantification of the target gene expression was calculated as previously described methods (Peng et al., 2021).

**Table 1:** Primers used for real-time PCR analysis.

Gene name	Primer sequence (5′ to 3′)
β-actin	F: GCTCTGTCGGCCTCTCAGG	R: CGTCGCACTTCATGATCGAG
TLR2	F: TCACTTGTCTAACTTATCATCCTCTTG	R: TCAGCGAAGGTGTCATTATTGC
TLR4	F: GCCATCGCTGCTAACATCATC	R: CTCATACTAAAGATACACCATCGG
FcRn	F: GGCGACGAGCACCACTACTG	R: AGCCGACCATGATTCCAACC
P65	F: GCATCCGTCGACAACTCTGA	R: CAGGTGTCAGCCCTTTAGGA

### Western blot

The total protein of jejunal mucosa and IPEC-J2 cells was extracted with protein extraction reagents (Thermo Fisher Scientific Inc., United States). Proteins were separated by 10% sodium dodecyl sulfate polyacrylamide gel electrophoresis (SDS-PAGE) and then electrotransferred to polyvinylidene difluoride (PVDF) membranes (BioRad, Hercules, CA, United States). Membranes were blocked and then incubated with the following primary antibodies overnight at 4°C: β-actin (Cell Signaling Technology, #4970), FcRn (Abclonal, A8544), NF-κB P65 (Cell Signaling Technology, #6956), and Phospho-NF-κB P65 (Ser536) (Cell Signaling Technology, #3033). After primary antibody incubation, membranes were washed and then incubated with HRP-conjugated anti-mouse or anti-rabbit IgG antibodies (Beyotime Biotechnology, Shanghai, China). Finally, quantified and digitally analyzed using the image J program (NIH).

### Transcriptomic analysis of jejunal mucosa

The total RNA was extracted from jejunal mucosa using Trizol according to the manufacturer’s instructions. Paired-end (PE) RNA-seq libraries were constructed using the Truseq mRNA-stranded RNA-Seq Library Prep Kit (Illumina, United States). The sequencing of the libraries with a read length of 2 × 150 bp and an insert size of 380 bp was performed on an Illumina HiSeq X Ten sequencing platform. The quality of the raw sequencing data was assessed and filtered with RNA-QC-Chain (Zhou et al., 2018), removing the adaptors, contaminations, and low-quality reads. The filtered reads were aligned to the reference genome of the *S. scrofa* (NCBI Accession No. GCF_000003025.6) using HISAT2 (v2.1.0) (Kim et al., 2019). The expression level of genes was estimated using fragments per kilobase per million mapped reads (FPKM) by StringTie (v1.3.6) (Pertea et al., 2015). We used DESeq to detect the differentially expressed genes (DEGs), which were defined as genes with|log2(FoldChange)| > 1 and adjusted *p* < 0.05 between the two groups. The Benjamini-Hochberg (BH) method-based false discovery rate (FDR) multiple test correction was applied to adjust the *p* value.

### Metagenomic analysis of jejunal microbiome

The total DNA from the jejunal content samples was extracted using QIAamp DNA Stool Mini Kit (Qiagen, Germany) following the manufacturer’s instructions. After the quality check, DNA was used to construct the PE libraries using NEBNext Ultr DNA Library Prep Kit for Illumina (NEB, United States). A total of 20 samples were successfully sequenced on Illumina HiSeq X Ten platform. The raw sequencing data were evaluated using FastQC and then trimmed by Fastp (v0.20.0) (Brown et al., 2017) to eliminate reads less than 50 bp, adapters, leading or trailing bases with Phred base quality (BQ) scores of <20, and fragments of every five bases with an average BQ score of <25. To obtain the taxonomical composition of the intestinal microbiome for each sample, the high-quality reads were annotated by MetaPhlAn2 (v2.6.0) with default settings (Truong et al., 2015). Alpha diversity was calculated using the QIIME and principal component analysis (PCA) were analyzed by R (v4.0.3) and QIIME. The top 50 taxons with significant differences in relative abundance between groups were clustered by R (v4.0.3).

### Phosphoproteomic analysis

**Figure 2:**
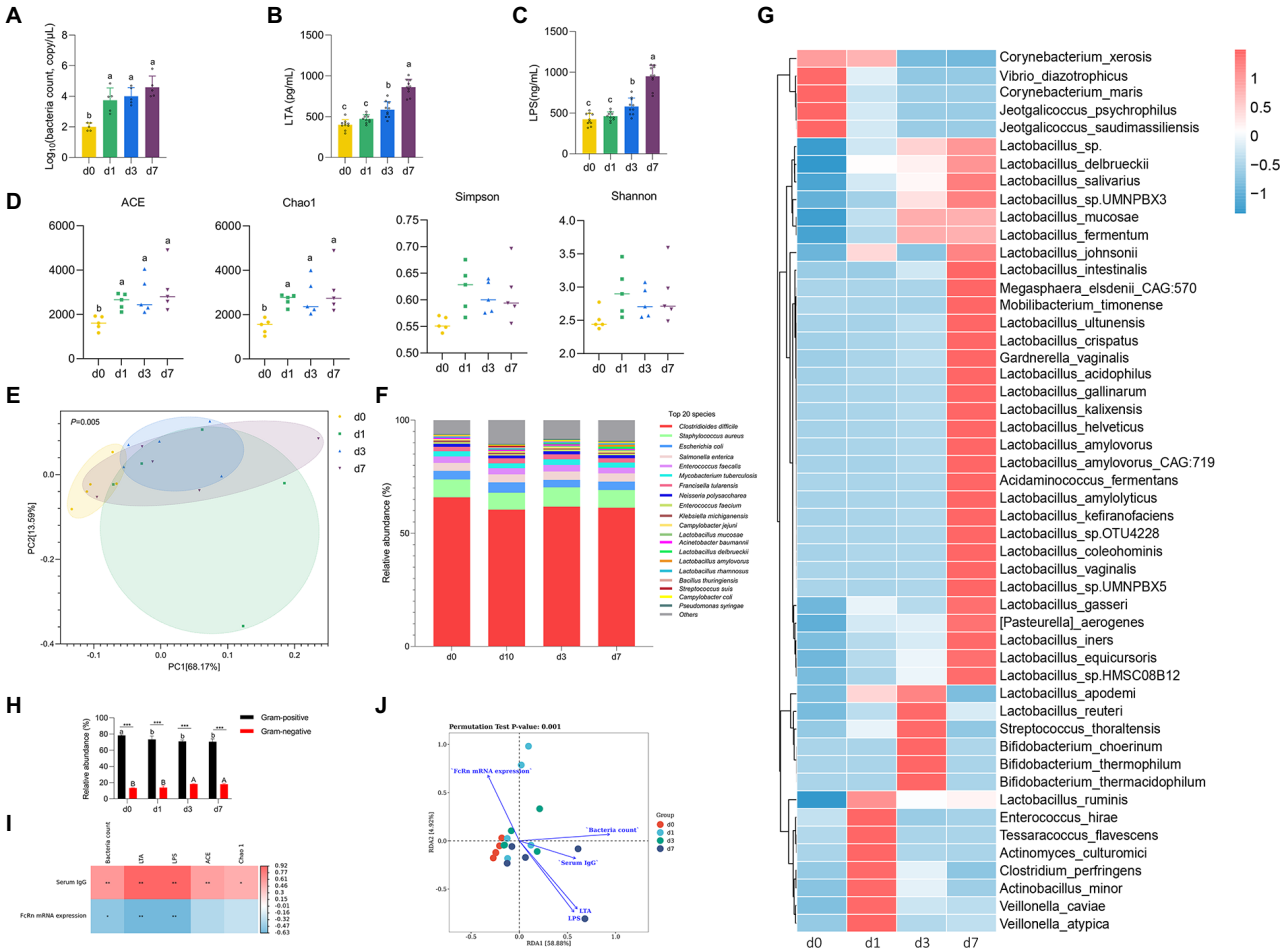
Dynamic changes of intestinal microbiota in piglets after birth. **(A)** Absolute quantitative analysis of jejunal microbiota in piglets at four time points (d 0, 1, 3, and 7). **(B,C)** LPS and LTA contents in jejunum of piglets at four time points (d 0, 1, 3, and 7). **(D)** Analysis of alpha-diversity in jejunal microflora at four time points (d 0, 1, 3, and 7). **(E)** Principal component analysis (PCA) of the structure of the jejunal microbiota at four time points (d 0, 1, 3, and 7). **(F)** The relative abundances of the jejunal microbiota at the specie levels at four time points (d 0, 1, 3, and 7). **(G)** The top 50 species level differential bacteria in jejunum of piglets at four time points (d 0, 1, 3, and 7). **(H)** Relative abundance of Gram-positive and Gram-negative bacteria in the top 20 species levels of jejunum of piglets at four time points (d 0, 1, 3, and 7). **(I)** Correlation analysis betwwen intestinal flora parameters and serum IgG levels and jejunal FcRn mRNA expression of piglets. **(J)** Redundancy analysis (RDA) between the top 50 jejunal bacteria in genus level and serum IgG content, jejunal LPS content, jejunal LTA content, total jejunal bacteria, and mRAN expression level of FcRn in jejunal mucosal tissue. Data were shown as means ± standard deviations. ^*^p < 0.05, ^**^p < 0.01, ^***^p < 0.001. Mean value without the common letter on data bar in each figure indicated that the difference was significant (*p* < 0.05).

There were two groups (*n* = 3 each group) of IPEC-J2 cells that were used for the phosphoproteomic analysis, one is LPS treatment group, treated with 1 μg/mL LPS for 2 h, and the other is LTA treatment group, treated with 2.5 μg/mL LTA for 2 h. SDT (4%SDS, 100 mM Tris–HCl, 1 mM DTT, pH7.6) buffer was used for sample lysis and protein extraction. The amount of protein was quantified with the BCA Protein Assay Kit (Bio-Rad, United States) following the manufacturer’s instructions. Protein digestion and phosphopeptides enrichment were performed as described previously (Wiśniewski et al., 2009). The enriched phosphopeptides was concentrated in vacuum and redissolved in 20 μL 0.1% formic acid solution for mass spectrometry analysis. Liquid chromatography–tandem mass spectrometry (LC–MS/MS) analysis was carried out by Applied Protein Technology Co., Ltd., Shanghai, China. The MS raw data for each sample were combined and searched using the Maxquant (1.5.2.8) software for identification and quantitation analysis. Phosphopeptides with the Fold Change >2 (up-regulated) or Fold Change <0.5 (down-regulated) were further applied to Student’s *t*-test at a univariate level to measure the significance, the *p*-values <0.05 were considered statistically significant (Figure 2A).

### Statistical analysis

Data are presented as means ± standard deviation (SD). A two-sided unpaired Student’s *t*-test with Benjamini–Hochberg correction was used to compare the two groups. The differences among the three groups were analyzed using one-way analysis of variance (ANOVA) followed by Duncan’s test. Significance was set at *p* < 0.05.

## Results

### The expression of FcRn promoted the absorption of IgG

Piglets which did not receive milk after parturition were recorded as d 0, and 24 h after suckling were recorded as d 1. As shown in Figure 1B, with the growth of piglets, the IgG levels in the stomach milk of piglets on d 1 were significantly higher than that on d 3 and d 7 (*p* < 0.05). On the contrary, the serum IgG content of piglets continued to increase from the d 1 of suckling to the peak on the d 7. The IgG content in serum of piglets on d 3 and d 7 was significantly higher than that on d 1 (*p* < 0.05). It is known that IgG in newborn piglets is derived entirely from breast milk, as shown in Figure 1C, the serum of piglets at d 0 contains no IgG. And FcRn is the only receptor for transporting IgG. We detected the mRNA expression and protein expression of FcRn in jejunal mucosa of piglets (Figures 1D,E). The trend of FcRn mRNA expression and protein expression was consistent, with the highest expression on the d 1 and then down-regulated. There was a significant difference between d 1 and d 3 and d 7 in FcRn mRNA expression and protein expression (*p* < 0.05), but the difference between d 3 and d 7 was not significant (*p* > 0.05). The mRNA expression of FcRn in jejunal mucosa of piglets was significantly different between d 0 and d 1 (*p* < 0.05), but no significant difference in protein expression (*p* > 0.05).

### Dynamic changes of intestinal microbiota in piglets after birth

**Figure 3:**
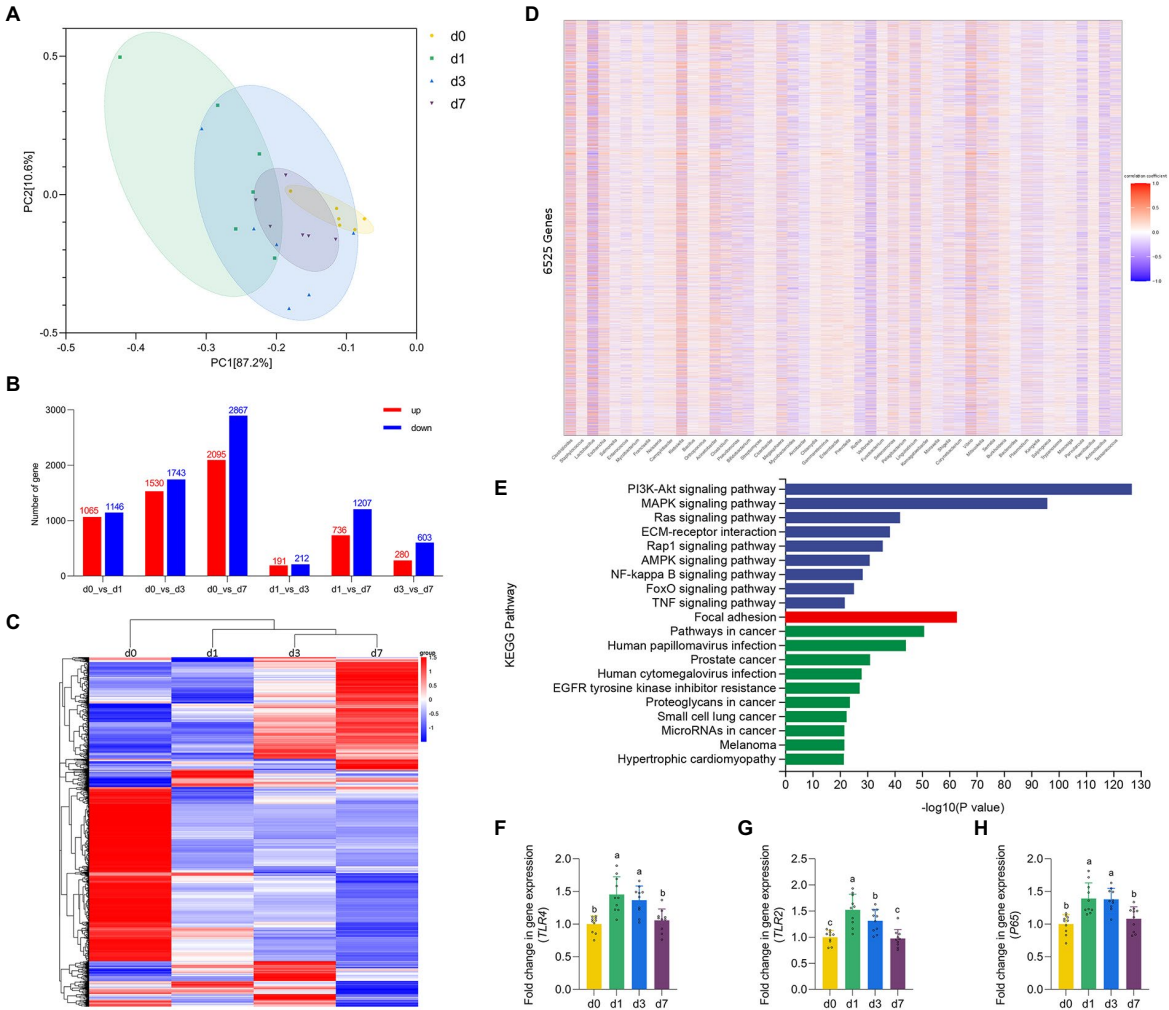
Dynamic gene expressions during intestinal development progression in piglets. **(A)** Principal component analysis (PCA) based on the gene expression in jejunal mucosa of piglets at four time points (d 0, 1, 3, and 7). **(B)** Histogram of differentially expressed genes in jejunal mucosa of piglets at four time points (d 0, 1, 3, and 7). **(C)** Cluster analysis results of differential expression of gene in jejunal mucosa of piglets at four time points (d 0, 1, 3, and 7). **(D)** Spearman correlation analysis between the top 50 species level differential bacteria screened in the metagenomic results and 6,525 defferent expreesed genes. **(E)** KEGG pathway enrichment analysis of genes highly associated with different bacteria. **(F–H)** The mRNA expression of TLR4, TLR2 and P65 in jejunal mucosa of piglets at four time points (d 0, 1, 3, and 7). Data were shown as means ± standard deviations. Mean value without the common letter on data bar in each figure indicated that the difference was significant (*p* < 0.05).

We performed absolute quantitative analysis of jejunal microbiota in piglets at four time points (d 0, 1, 3, and 7). As shown in Figure 3A, the amount of jejunal bacteria at d 0 was significantly lower than that at the other three time points (d 1, 3, and 7) (*p* < 0.05), while there was no significant difference among the other three time points (*p* > 0.05). The results of LPS content and LTA content in jejunum were consistent (Figures 3B,C). From d 0 to d 7, the contents of LPS and LTA in jejunum of piglets increased gradually. The contents of LPS and LTA in jejunum of piglets were not significantly different between d 0 and d 1 (*p* > 0.05), but d 0 and d 1 were significantly different from d 3 and d 7 (*p* < 0.05), respectively, and there were also significant differences between d 3 and d 7 (*p* < 0.05). To further clarify the colonization rules of intestinal microbiota in piglets, metagenomic sequencing was performed on intestinal microbiota at four time points. The Chao 1 and ACE indices that estimate microbial richness, and the Shannon and Simpson diversity indices which reflects species biodiversity, were calculated to evaluate the alpha diversity (Figure 3D). The species richness (Chao 1 and ACE) of d 0 was significantly lower than that of other three time points (d 1, 3, and 7) (*p* < 0.05). The results of PCA showed that jejunal microbiota of piglets at the four time points did not completely overlap, indicating that the jejunum of piglets was colonized with different bacteria at the four time points (Figure 3E). Besides, we analyzed the taxonomic composition and top 50 differential bacteria composition of jejunum flora of piglets at four time points (Figures 3F,G). At species level, the dominant bacteria in jejunum of piglets was *Clostridioides difficile* both at four time points. Except for *Lactobacillus mucosae*, *Lactobacillus delbrueckii*, and *Lactobacillus amylovorus*, there was no significant difference in the relative abundance of other bacteria in jejunum of piglets at four time points. The analysis of top 50 differential bacteria composition also reflects the colonization rule of piglets jejunal microbiota at four time points. By the 7th day after birth, *Lactobacillus* gradually became the dominant bacteria in jejunal of piglets. In addition, we calculated the proportion of Gram-positive bacteria and Gram-negative bacteria in the top 20 species levels of jejunum of piglets (Figure 3H). The results showed that the relative abundance of Gram-positive bacteria in jejunum significantly decreased and the relative abundance of Gram-negative bacteria significantly increased with the increase of age of piglets (*p* < 0.05). And the proportion of Gram-positive bacteria in jejunum of piglets was always higher than that of Gram-negative bacteria at four time points (*p* < 0.05).

### Correlation analysis of IgG absorption and intestinal flora in piglets

We analyzed the correlation between serum IgG content and jejunal mucosal FcRn mRNA expression with total jejunal bacteria, jejunal LPS content, jejunal LTA content and jejunal microbiota alpha diversity index (Chao1 and ACE) at 4 time points, respectively. As shown in Figure 3I, the content of serum IgG had significantly positive correlation with total jejunal bacteria, jejunal LPS content, jejunal LTA content and jejunal microbiota alpha diversity index (Chao1 and ACE) (*p* < 0.05), and the expression of FcRn mRNA in jejunum had significantly negative correlation with total jejunal bacteria, jejunal LPS content and jejunal LTA content (*p* < 0.05). Obviously, the content of LPS and LTA in jejunum had the strongest correlation with serum IgG content and jejunal FcRn mRNA expression (*p* < 0.01). Moreover, we performed redundancy analysis (RDA) between the top 50 jejunal bacteria in genus level and serum IgG content, jejunal LPS content, jejunal LTA content, total jejunal bacteria, and mRAN expression level of FcRn in jejunal mucosal tissue. The results of RDA showed that the microflora composition in jejunum of piglets at four time points had no significant difference in genus level. Furthermore, the interaction between jejunum microbial composition and serum IgG content, jejunal LPS content, jejunal LTA content, total jejunal bacteria, and mRAN expression level of FcRn in jejunal mucosal tissue was highly significant (*p* = 0.001) (Figure 3J).

### Dynamic gene expressions during intestinal development progression in piglets

**Figure 4:**
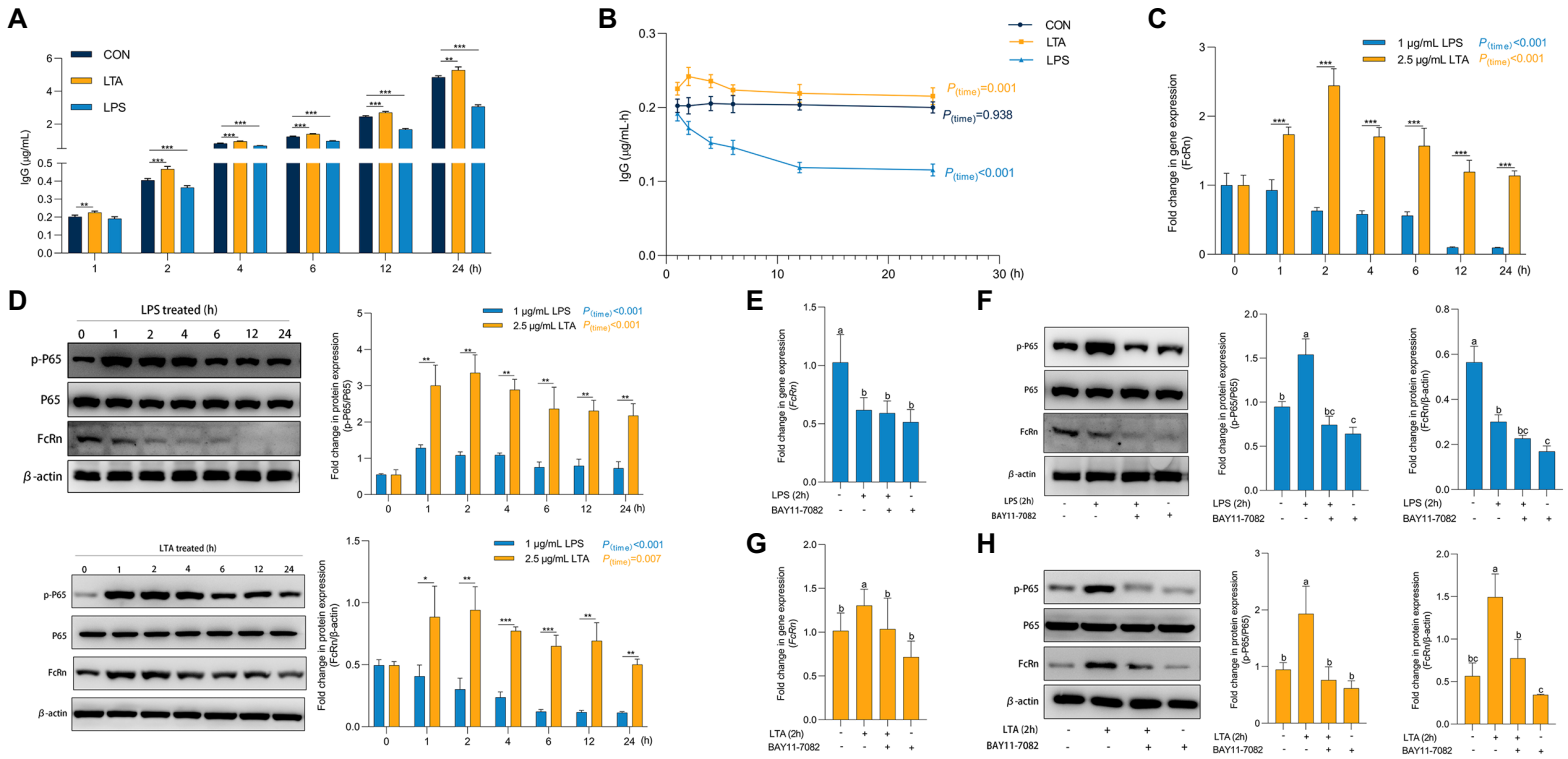
Role of P65 phosphorylation in transmembrane transport of IgG. **(A,B)** Transmembrane transport of IgG after LPS and LTA stimulation. **(C)** The mRNA expression of FcRn after LPS and LTA stimulation. **(D)** The protein expression of FcRn, P65, and P65 phosphorylation after LPS and LTA stimulation. **(E–H)** The mRNA expression of FcRn and the protein expression of FcRn, P65 and P65a phosphorylation after blocking NF-κB signaling pathway and then stimulated by LPS and LTA. Data were shown as means ± standard deviations. ^*^p < 0.05, ^**^p < 0.01, ^***^p < 0.001. Mean value without the common letter on data bar in each figure indicated that the difference was significant (*p* < 0.05).

The results of PCA based on the gene expression of four time points reflected the degree of differences in the genetic profiles of piglets at d 0, 1, 3, and 7 (Figure 4A). Through the traditional *t*-test, the differences between the combinations of any two time periods were found (Figure 4B), and finally the list of 6,525 DEGs was combined. Then, after performing unsupervised hierarchical clustering based on the identified DEGs (Figure 4C), we found that the samples at the four time points (d0, d1, d3, d7) could be roughly clustered into three independent groups (d 0 as one group, d 1 as one group, d 3 and d 7 as one group). This suggested that d 1 may be the key point of intestinal function transformation in piglets. Similarly, d 1 is a key time point for intestinal flora colonization. Therefore, we conducted Spearman correlation analysis between the top 50 species level differential bacteria screened in the metagenomic results and 6,525 DEGs (Figure 4D). We screened 6,525 DEGs with |correlation coefficient| > 0.6 and adjusted *p* < 0.05, and performed KEGG pathway enrichment analysis on the eligible genes (Figure 4E). The results showed that genes strongly associated with differential bacteria were enriched into three categories: Environmental Information Processing, Cellular Processes and Human Diseases, including the NF-κB signaling pathway. Finally, we verified the expression levels of some genes of the NF-κB signaling pathway (Figures 4F–H). The gene expression of TLR2, TLR4 and P65 in the jejunum of piglets were significantly upregulated at d 1 when compared with d 0 (*p* < 0.05). Besides, all three genes were downregulated from d 1 to d 7, and all three genes mRNA expression were significantly inhibited at d 7 versus the d 1 (*p* < 0.05).

### NF-κB signaling pathway mediates IgG transmembrane transport through FcRn

**Figure 5:**
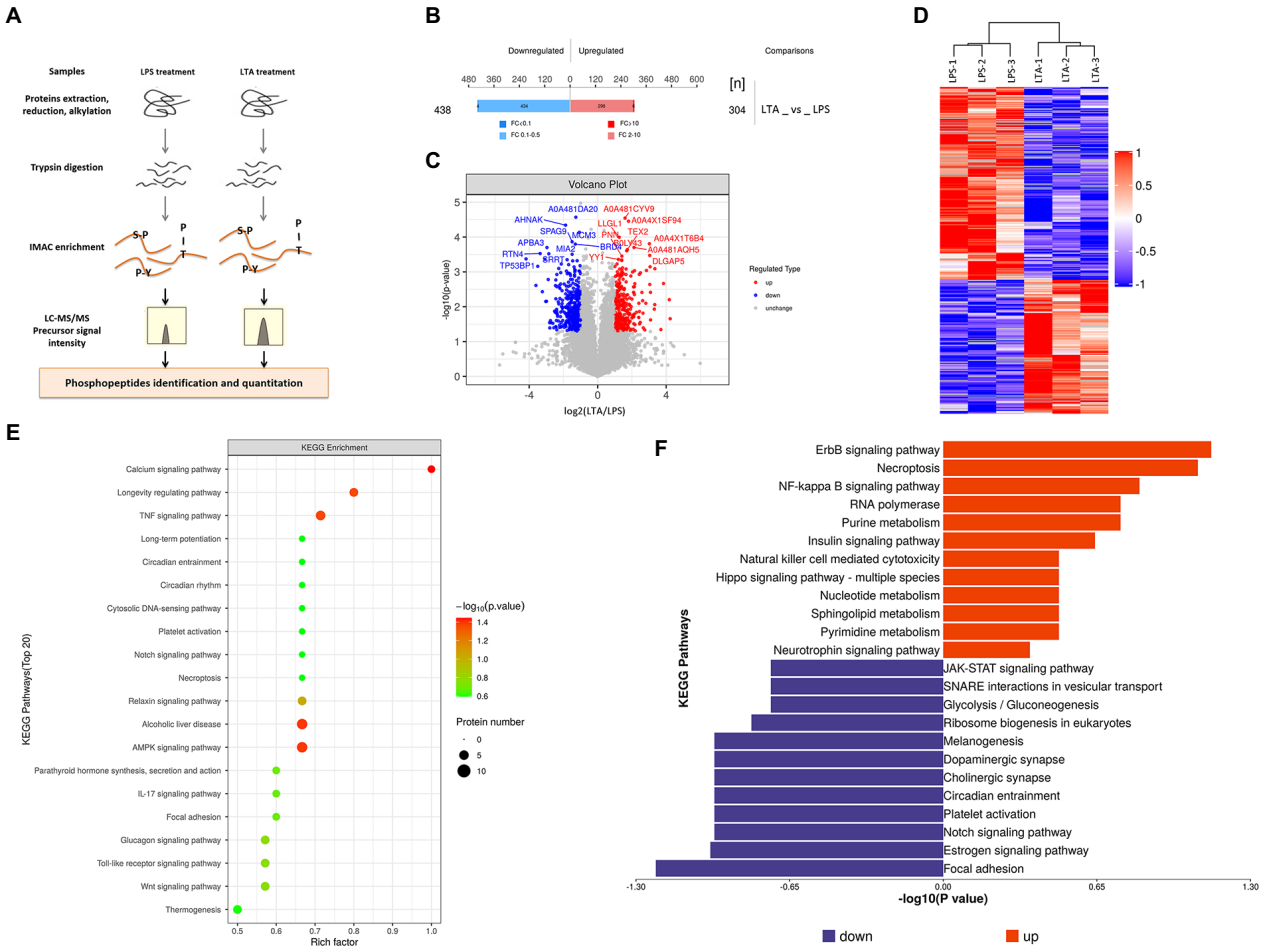
Phosphorylated modification of proteins after LPS and LTA stimulation. **(A)** Overview of the workflow of phosphorylated proteomics. **(B)** Histogram of quantitative differences in phosphorylated peptides between the LPS and LTA groups. **(C)** Volcano plot shows the different phosphorylated peptide between the LPS and LTA groups. **(D)** Cluster analysis results of differential expression of phosphorylated peptide between LTA group and LPS group. **(E,F)** Enrichment results of KEGG pathway corresponding to phosphorylated peptides with differential expression between the LPS and LTA groups.

As shown in Supplementary Figure S1, when the concentration of LPS and LTA were 1 μg/mL and 2.5 μg/mL, respectively, the cell viability and cell barrier function were not affected by 24 h incubation with cells. Thus, we incubated IPEC-J2 with 1 μg/mL LPS and 2.5 μg/mL LTA for 24 h, respectively, to explore the effects of cells treated with LPS or LTA on IgG transmembrane transport (Figures 5A,B). Obviously, from the second hour onwards, the IgG content in transwell lower chamber and IgG transmembrane transport rate at each time point (2, 4, 6, 12, 24 h) in LPS treated group were always significantly lower than those in CON group (*p* < 0.05). In contrast, the LTA treated group significantly increased the content of IgG in transwell lower chamber transported from the upper chamber compared to CON group at whole time (1, 2, 4, 6, 12, 24 h) (*p* < 0.05). As shown in Figure 5C, with the accumulation of incubation time, LPS treatment significantly inhibited the mRNA expression of FcRn (*p* < 0.001), while LTA treatment significantly enhanced the mRNA expression of FcRn (*p* < 0.001). Apparently, the mRNA expression of FcRn in LTA group was significantly up-regulated versus the LPS group at whole time (1, 2, 4, 6, 12, 24 h) (*p* < 0.001). Moreover, Western blot analysis showed that LPS and LTA treatment both activated NF-κB signaling pathway and promoted the phosphorylation of P65 (*p* < 0.001). The activation of NF-κB signaling pathway by LTA was significantly stronger than that by LPS (*p* < 0.01). Consistent with the results of qPCR, LPS treatment remarkably inhibited the protein level of FcRn expression (*p* < 0.001) while LTA treatment markedly promoted the protein level of FcRn expression (*p* = 0.007) compared with the cells without any treatment. Apparently, the protein level of FcRn expression in LTA group was significantly increased versus the LPS group at whole time (1, 2, 4, 6, 12, 24 h; *p* < 0.05; Figure 5D). Therefore, to explore whether the down-regulation and up-regulation of FcRn induced by LPS and LTA were related to the NF-κB signaling pathway, NF-κB inhibitor BAY11-7082 was used to pretreat IPEC-J2 cells before stimulation with 1 μg/mL LPS or 2.5 μg/mL LTA. We found that blocking NF-κB signaling pathway decreased mRNA and protein expressions of FcRn induced by LTA, but it did not affect the down-regulation of mRNA and protein expressions of FcRn induced by LPS (Figures 5E–H).

### Differences in phosphorylated proteome between LPS and LTA treated cells

Both LPS and LTA treated cells activated the NF-κB pathway, but LPS inhibited the expression of FcRn while LTA promoted the expression of FcRn. In order to explore the reason for the difference between LPS and LTA treatment groups, phosphorylated proteome was detected in cells treated with LPS or LTA (Figure 2). A total of 742 differentially modified peptides were identified by LPS and LTA treatment groups. Compared with LPS treatment group, 304 peptides were up-regulated and 438 peptides were down-regulated in LTA treatment group (*p* < 0.05; Figure 2B). Significant differences in phosphorylated modified peptides between LPS and LTA treatment groups were visualized in the form of a volcano plot (Figure 2C). Hierarchical clustering algorithm was used to group and classify the differentially expressed phosphorylated peptides in LPS and LTA treatment groups. The proteins corresponding to the significantly differentially expressed phosphorylated peptides obtained can effectively separate the comparison groups, indicating that the screening of differentially expressed phosphorylated peptides can represent the influence of biological treatment on samples (Figure 2D). Next, we performed KEGG pathway enrichment analysis on the proteins corresponding to the differentially expressed phosphorylated peptides screened, and the results showed that significant changes occurred in important pathways such as calcium signaling pathway, AMPK signaling pathway, alcoholic liver disease, TNF signaling pathway, longevity regulating pathway (Figure 2E). In addition, KEGG signaling pathway enrichment of differentially expressed phosphorylated peptides showed that NF-κB signaling pathway was significantly up-regulated in LTA treatment group compared with LPS treatment group (*p* < 0.05; Figure 2F).

## Discussion

How to improve the passive immune ability and maternal antibody level of piglets by nutritional means is the key to effectively prevent intestinal infection of piglets and improve the survival rate and production efficiency of piglets. The main immunoglobulin in colostrum within 3 days after parturition is IgG. Therefore, the limited absorption of IgG in breast milk is the main reason for the low innate passive immunity of piglets. As the only receptor for specific IgG transport, FcRn has attracted much attention. FcRn has more recently been shown to express in a variety of mammalian species (Ward and Ober, 2009). Meanwhile, several studies have reported the distribution, function, and regulation of human and rodent FcRn expression (Dickinson et al., 1999; Jiang et al., 2004; Liu et al., 2007; Tian et al., 2014). In this study, we provided evidence that the expression of FcRn in the gut of piglets peaked on postnatal d 1, and thereafter FcRn expression gradually decreased with the piglets age, consistent with previous studies (Mayer et al., 2002). On the other hand, the IgG content of breast milk changed rapidly with the passage of postpartum time, decreasing by 30% within 3 h after delivery and by nearly 90% at 24 h after delivery (Kielland et al., 2015; Hue et al., 2021). Therefore, targeted regulation of FcRn molecule during the IgG-rich period of breast milk may be an effective measure to improve IgG absorption efficiency in piglets. But the molecular mechanisms and nutritional measures of FcRn regulation are still rarely reported.

In our present study, the results shown that the serum of piglets that did not drink breast milk at birth did not contain IgG, but the expression of FcRn in the intestinal tract of piglets maintained a high level. This is determined inborn so that piglets exposed to breast milk can quickly absorb IgG in breast milk. It has not been reported whether the regulation of intestinal FcRn expression in piglets can be achieved during the embryonic period. At the time point of d 1, the expression of FcRn in the intestine of piglets increased rapidly and reached the peak within 7 days. Meanwhile, the content of IgG in serum of piglets also increased sharply. From d 3 to d 7, the expression level of FcRn in the intestinal tract of piglets began to be significantly down-regulated, and the content of IgG in serum of piglets also remained stable and did not increase. This indicates that the increase of FcRn expression promotes the absorption of IgG, which is also limited when FcRn expression is suppressed.

Several studies have shown that the colonization of gut microbiota in early life can affect the physiological function of the host gut, especially the early development of the host immune system (Milani et al., 2017; Zhuang et al., 2019; Al and Eberl, 2020). The immune system of piglets is not established after birth, and it does not start until 7–8 days after birth. During this period, the main source of immune material of piglets was IgG obtained from the mother. Thus, we speculate that the colonization of intestinal flora of newborn piglets may affect the absorption of IgG to a certain extent. In this work, the total bacteria content in the intestine of piglets increased rapidly and the diversity of bacteria increased significantly from d 1. Moreover, the jejunum flora composition of piglets at d 1 and d 0 time points was significantly different. The above results showed that intestinal flora began to colonize from d 1, and it was during this process that the expression level of intestinal FcRn increased significantly. This may mean that during the colonization of intestinal flora, some components stimulate the expression of FcRn in some way to promote the transport of IgG in piglets. In Helicobacter pylori-infected mice, the expression of FcRn in the gastric epithelium is upregulated to facilitate the transport of IgG from blood to gastric fluid (Ben et al., 2012). Similarly, Li et al. (2021) found that adding the heat inactivated Clostridium butyricum CB1 into porcine small intestinal epithelial cells promoted the expression of FcRn on the cell membrane. These studies provide direct evidence that epithelial expression of FcRn is able to link luminal and/or epithelial infectious exposures with systemic immune activation. In other words, the intervention of antigenic substances does promote the expression of FcRn and thus regulate the transmembrane transport of IgG. Consistent with previous studies, our results also showed that the expression of FcRn in jejunal mucosa of piglets was significantly correlated with the intestinal flora parameters.

In order to clarify the specific mechanism of intestinal flora participating in the regulation of FcRn, we conducted correlation analysis between the differential bacteria screened by metagenomes and the DEGs screened by the transcriptome, and selected genes with high correlation coefficients for KEGG pathway enrichment analysis. We identified the NF-κB signaling pathway, which is closely related to microbes and immunity. Previous studies have shown that there are five NF-κB (p65) binding sites in the FcRn promoter region, and overexpression or agonist activation of the NF-κB signaling pathway can significantly improve the expression of FcRn (Guo et al., 2016a,b). To verify our hypothesis that intestinal flora regulates FcRn expression and mediates IgG transport through the NF-κB signaling pathway, we conducted *in vitro* cell experiments. The results showed that intestinal antigens mediated the transmembrane transport of IgG by regulating the expression of FcRn through the NF-κB signaling pathway. Interestingly, both LTA and LPS activated the NF-κB signaling pathway, but LTA promoted FcRn expression while LPS inhibited FcRn expression, which is inconsistent with the results reported by Cervenak that LPS promotes FcRn expression in bovine aorta endothelial cells (Cervenak et al., 2013). We compared the results of phosphorylated proteome in LPS-treated and LTA-treated cells, and found that the activation intensity of NF-κB signaling pathway in LTA stimulated cells was significantly higher than that of LPS. On the other hand, the activation degree of JAK–STAT signaling pathway in LPS group was significantly higher than that in LTA group. Studies have shown that activation of JAK–STAT signaling pathway could inhibit the expression of FcRn (Liu et al., 2008). Therefore, we speculated that LPS treatment activated the NF-κB signaling pathway but inhibited the expression of FcRn because of the antagonistic effect of JAK–STAT and NF-κB signaling pathway in regulating FcRn. LPS stimulated JAK–STAT signaling pathway was significantly more activated than NF-κB signaling pathway, thus ultimately inhibiting FcRn expression.

## Conclusion

**Figure 6:**
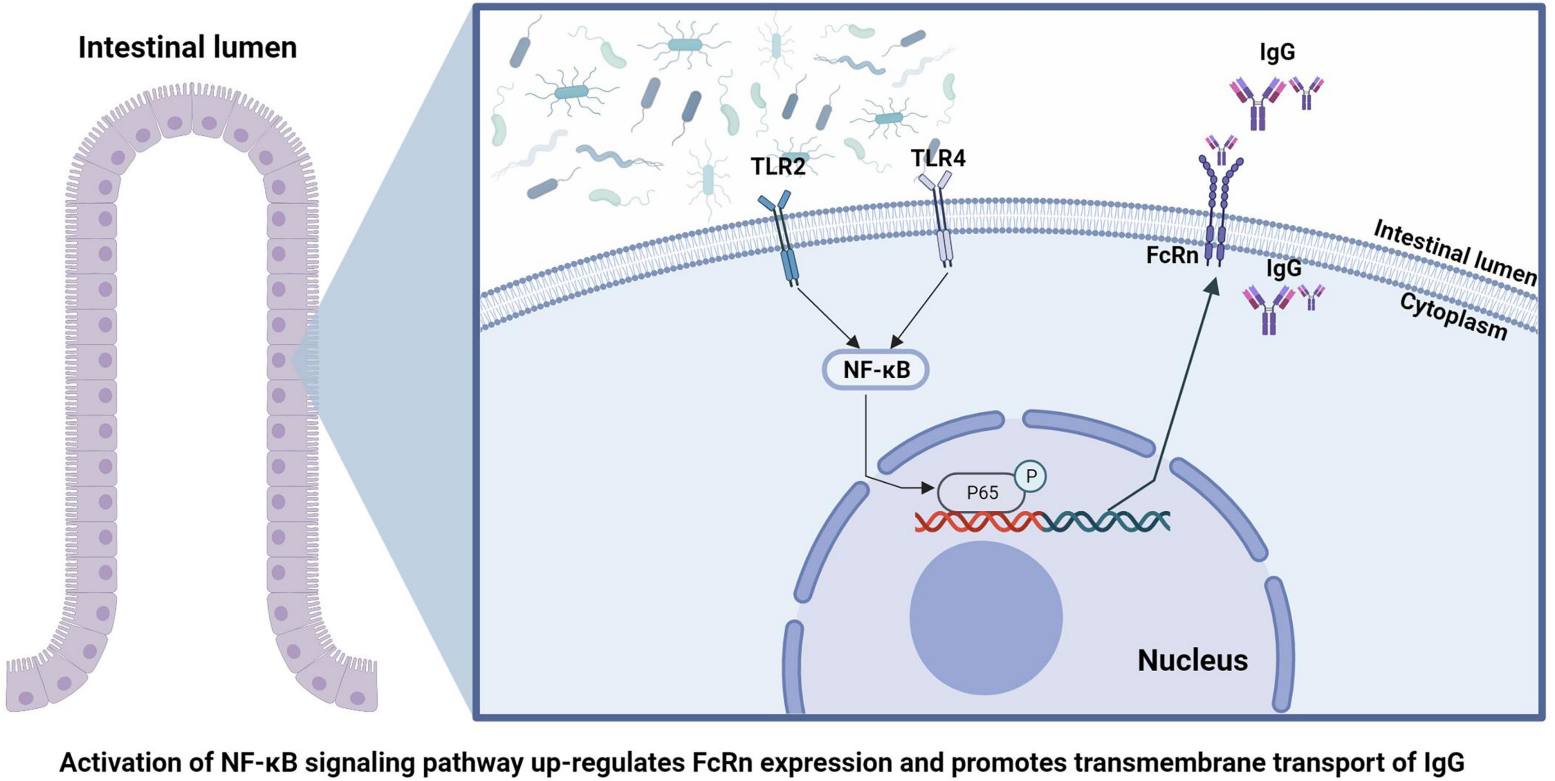
Schematic summary of the findings in this study.

In conclusion, by comparing the absorption of IgG and colonization of intestinal flora of piglets at four time points after birth, we found that the early colonization of intestinal flora can affect the absorption of IgG in the intestine of piglets. The absorption of newborn piglets IgG depends on the expression of FcRn in the gut, and the gut flora is involved in regulating the expression of FcRn through the NF-κB signaling pathway (Figure 6). This suggests that we can promote the absorption of breast milk IgG by means of early intestinal flora intervention, so as to improving the passive immune function of newborn piglets and ensure the survival rate of newborn piglets.

## Data availability statement

The datasets presented in this study can be found in online repositories. The names of the repository/repositories and accession number(s) can be found at: NCBI – PRJNA918967 and PRJNA918527.

## Ethics statement

The animal study was reviewed and approved by the Institutional Animal Care and Use Committee of the Hunan Agricultural University.

## Author contributions

FP designed the experiment, conducted the research, and wrote the manuscript. HZ and ZS revised the article. XH analyzed the data. All authors contributed to the article and approved the submitted version.

## Funding

The authors are thankful to the Project supported by the National Natural Science Foundation of China (Grant No: 31902171).

## Conflict of interest

The authors declare that the research was conducted in the absence of any commercial or financial relationships that could be construed as a potential conflict of interest.

## Publisher’s note

All claims expressed in this article are solely those of the authors and do not necessarily represent those of their affiliated organizations, or those of the publisher, the editors and the reviewers. Any product that may be evaluated in this article, or claim that may be made by its manufacturer, is not guaranteed or endorsed by the publisher.

## Supplementary material

The Supplementary material for this article can be found online at: https://www.frontiersin.org/articles/10.3389/fmicb.2023.1136513/full#supplementary-material

## References

[ref1] Al N. Z. Eberl G. (2020). Imprinting of the immune system by the microbiota early in life. Mucosal Immunol. 13, 183–189. doi: 10.1038/s41385-020-0257-y, PMID: 31988466

[ref2] Andraud M. Fablet C. Renson P. Eono F. Mahé S. Bourry O.. (2018). Estimating parameters related to the lifespan of passively transferred and vaccine-induced porcine reproductive and respiratory syndrome virus type I antibodies by modeling field data. Front. Vet. Sci. 5:9. doi: 10.3389/fvets.2018.00009, PMID: 29435455 PMC5796902

[ref3] Bandrick M. Ariza-Nieto C. Baidoo S. K. Molitor T. W. (2014). Colostral antibody-mediated and cell-mediated immunity contributes to innate and antigen-specific immunity in piglets. Dev. Comp. Immunol. 43, 114–120. doi: 10.1016/j.dci.2013.11.005, PMID: 24252519 PMC3902642

[ref4] Ben S. Y. Yoshida M. Nishiumi S. Tanaka H. Mimura T. Nobutani K.. (2012). Neonatal Fc receptor for IgG (FcRn) expressed in the gastric epithelium regulates bacterial infection in mice. Mucosal Immunol. 5, 87–98. doi: 10.1038/mi.2011.53, PMID: 22089027 PMC3964614

[ref5] Bournazos S. Ravetch J. V. (2017). Diversification of igg effector functions. Int. Immunol. 29, 303–310. doi: 10.1093/intimm/dxx025, PMID: 28472280 PMC5890892

[ref6] Brown J. Pirrung M. McCue L. A. (2017). FQC Dashboard: integrates FastQC results into a web-based, interactive, and extensible FASTQ quality control tool. Bioinformatics 33, 3137–3139. doi: 10.1093/bioinformatics/btx373, PMID: 28605449 PMC5870778

[ref7] Cabrera R. A. Lin X. Campbell J. M. Moeser A. J. Odle J. (2012). Influence of birth order, birth weight, colostrum and serum immunoglobulin g on neonatal piglet survival. J. Anim. Sci. Biotechnol. 3:42. doi: 10.1186/2049-1891-3-42, PMID: 23259926 PMC3541264

[ref8] Cervenak J. Doleschall M. Bender B. Mayer B. Schneider Z. Doleschall Z.. (2013). NFκB induces overexpression of bovine FcRn: a novel mechanism that further contributes to the enhanced immune response in genetically modified animals carrying extra copies of FcRn. MAbs 5, 860–871. doi: 10.4161/mabs.26507, PMID: 24492342 PMC3896600

[ref9] Czech A. Grela E. R. Mokrzycka A. Pejsak Z. (2010). Efficacy of mannanoligosaccharides additive to sows diets on colostrum, blood immunoglobulin content and production parameters of piglets. Pol. J. Vet. Sci. 13, 525–531, PMID: . 21033568

[ref10] D'Hooghe L. Chalmers A. D. Heywood S. Whitley P. (2017). Cell surface dynamics and cellular distribution of endogenous FcRn. PLoS One 12:e182695. doi: 10.1371/journal.pone.0182695, PMID: 28817705 PMC5560688

[ref11] Dickinson B. L. Badizadegan K. Wu Z. Ahouse J. C. Zhu X. Simister N. E.. (1999). Bidirectional fcrn-dependent igg transport in a polarized human intestinal epithelial cell line. J. Clin. Invest. 104, 903–911. doi: 10.1172/JCI6968, PMID: 10510331 PMC408555

[ref12] Guo J. Li F. He Q. Jin H. Liu M. Li S.. (2016a). Neonatal fc receptor-mediated igg transport across porcine intestinal epithelial cells: potentially provide the mucosal protection. DNA Cell Biol. 35, 301–309. doi: 10.1089/dna.2015.3165, PMID: 26982157

[ref13] Guo J. Li F. Qian S. Bi D. He Q. Jin H.. (2016b). TGEV infection up-regulates FcRn expression via activation of NF-κB signaling. Sci. Rep. 6:32154. doi: 10.1038/srep32154, PMID: 27555521 PMC4995372

[ref14] Hue D. T. Skirving R. Chen T. Williams J. L. Bottema C. Petrovski K. (2021). Colostrum source and passive immunity transfer in dairy bull calves. J. Dairy Sci. 104, 8164–8176. doi: 10.3168/jds.2020-19318, PMID: 33865574

[ref15] Inoue R. Yajima T. Tsukahara T. (2017). Expression of TLR2 and TLR4 in murine small intestine during postnatal development. Biosci. Biotechnol. Biochem. 81, 350–358. doi: 10.1080/09168451.2016.1254534, PMID: 27838962

[ref16] Jiang L. Wang J. Solorzano-Vargas R. S. Tsai H. V. Gutierrez E. M. Ontiveros L. O.. (2004). Characterization of the rat intestinal fc receptor (FcRn) promoter: transcriptional regulation of FcRn gene by the Sp family of transcription factors. Am. J. Physiol. Gastroint. Liver Physiol. 286, G922–G931. doi: 10.1152/ajpgi.00131.2003, PMID: 15132949

[ref17] Kielland C. Rootwelt V. Reksen O. Framstad T. (2015). The association between immunoglobulin g in sow colostrum and piglet plasma. J. Anim. Sci. 93, 4453–4462. doi: 10.2527/jas.2014-8713, PMID: 26440345

[ref18] Kim D. Paggi J. M. Park C. Bennett C. Salzberg S. L. (2019). Graph-based genome alignment and genotyping with HISAT2 and HISAT-genotype. Nat. Biotechnol. 37, 907–915. doi: 10.1038/s41587-019-0201-4, PMID: 31375807 PMC7605509

[ref19] Kliwinski C. Cooper P. R. Perkinson R. Mabus J. R. Tam S. H. Wilkinson T. M.. (2013). Contribution of FcRn binding to intestinal uptake of igg in suckling rat pups and human FcRn-transgenic mice. Am. J. Physiol. Gastroint. Liver Physiol. 304, G262–G270. doi: 10.1152/ajpgi.00340.2012, PMID: 23220220

[ref20] Lauritsen K. T. Hagedorn-Olsen T. Jungersen G. Riber U. Stryhn H. Friis N. F.. (2017). Transfer of maternal immunity to piglets is involved in early protection against mycoplasma hyosynoviae infection. Vet. Immunol. Immunopathol. 183, 22–30. doi: 10.1016/j.vetimm.2016.12.002, PMID: 28063473

[ref21] Li C. Cao R. Qian S. Qiao C. Liu X. Zhou Z.. (2021). Clostridium butyricum CB1 up-regulates FcRn expression via activation of TLR2/4-NF-κB signaling pathway in porcine small intestinal cells. Vet. Immunol. Immunopathol. 240:110317. doi: 10.1016/j.vetimm.2021.110317, PMID: 34461425

[ref22] Liu X. Ye L. Bai Y. Mojidi H. Simister N. E. Zhu X. (2008). Activation of the JAK/STAT-1 signaling pathway by IFN-gamma can down-regulate functional expression of the MHC class I-related neonatal Fc receptor for IgG. J. Immunol. 181, 449–463. doi: 10.4049/jimmunol.181.1.449, PMID: 18566411 PMC2667120

[ref23] Liu X. Ye L. Christianson G. J. Yang J. Q. Roopenian D. C. Zhu X. (2007). NF-kappaB signaling regulates functional expression of the MHC class I-related neonatal Fc receptor for IgG via intronic binding sequences. J. Immunol. 179, 2999–3011. doi: 10.4049/jimmunol.179.5.2999, PMID: 17709515 PMC2667116

[ref24] Mayer B. Zolnai A. Frenyó L. V. Jancsik V. Szentirmay Z. Hammarström L.. (2002). Redistribution of the sheep neonatal fc receptor in the mammary gland around the time of parturition in ewes and its localization in the small intestine of neonatal lambs. Immunology 107, 288–296. doi: 10.1046/j.1365-2567.2002.01514.x, PMID: 12423304 PMC1782797

[ref25] Milani C. Duranti S. Bottacini F. Casey E. Turroni F. Mahony J.. (2017). The first microbial colonizers of the human gut: composition, activities, and health implications of the infant gut microbiota. Microbiol. Mol. Biol. Rev. 81:e00036-17. doi: 10.1128/MMBR.00036-17, PMID: 29118049 PMC5706746

[ref26] Mortensen S. A. Sander B. Jensen R. K. Pedersen J. S. Golas M. M. Jensenius J. C.. (2017). Structure and activation of c1, the complex initiating the classical pathway of the complement cascade. Proc. Natl. Acad. Sci. U. S. A. 114, 986–991. doi: 10.1073/pnas.1616998114, PMID: 28104818 PMC5293073

[ref27] Peng F. Zhang H. He X. Song Z. (2021). Effects of ursolic acid on intestinal health and gut bacteria antibiotic resistance in mice. Front. Physiol. 12:650190. doi: 10.3389/fphys.2021.650190, PMID: 34122127 PMC8195277

[ref28] Pertea M. Pertea G. M. Antonescu C. M. Chang T. C. Mendell J. T. Salzberg S. L. (2015). StringTie enables improved reconstruction of a transcriptome from RNA-seq reads. Nat. Biotechnol. 33, 290–295. doi: 10.1038/nbt.3122, PMID: 25690850 PMC4643835

[ref29] Pierzynowska K. Woliński J. Weström B. Pierzynowski S. G. (2020). Maternal immunoglobulins in infants-are they more than just a form of passive immunity? Front. Immunol. 11:855. doi: 10.3389/fimmu.2020.00855, PMID: 32508816 PMC7248395

[ref30] Round J. L. Lee S. M. Li J. Tran G. Jabri B. Chatila T. A.. (2011). The toll-like receptor 2 pathway establishes colonization by a commensal of the human microbiota. Science 332, 974–977. doi: 10.1126/science.1206095, PMID: 21512004 PMC3164325

[ref31] Tian Z. Sutton B. J. Zhang X. (2014). Distribution of rat neonatal fc receptor in the principal organs of neonatal and pubertal rats. J. Recept. Signal Transduct. Res. 34, 137–142. doi: 10.3109/10799893.2013.865745, PMID: 24303938

[ref32] Truong D. T. Franzosa E. A. Tickle T. L. Scholz M. Weingart G. Pasolli E.. (2015). Metaphlan2 for enhanced metagenomic taxonomic profiling. Nat. Methods 12, 902–903. doi: 10.1038/nmeth.3589, PMID: 26418763

[ref33] Ward E. S. Ober R. J. (2009). Chapter 4: multitasking by exploitation of intracellular transport functions the many faces of FcRn. Adv. Immunol. 103, 77–115. doi: 10.1016/S0065-2776(09)03004-1, PMID: 19755184 PMC4485553

[ref34] Wiśniewski J. R. Zougman A. Nagaraj N. Mann M. (2009). Universal sample preparation method for proteome analysis. Nat. Methods 6, 359–362. doi: 10.1038/nmeth.1322 19377485

[ref35] Zeng M. Y. Cisalpino D. Varadarajan S. Hellman J. Warren H. S. Cascalho M.. (2016). Gut microbiota-induced immunoglobulin g controls systemic infection by symbiotic bacteria and pathogens. Immunity 44, 647–658. doi: 10.1016/j.immuni.2016.02.006, PMID: 26944199 PMC4794373

[ref36] Zhou Q. Su X. Jing G. Chen S. Ning K. (2018). RNA-QC-chain: comprehensive and fast quality control for RNA-Seq data. BMC Genomics 19:144. doi: 10.1186/s12864-018-4503-6, PMID: 29444661 PMC5813327

[ref37] Zhuang L. Chen H. Zhang S. Zhuang J. Li Q. Feng Z. (2019). Intestinal microbiota in early life and its implications on childhood health. Genom. Proteomics Bioinformatics. 17, 13–25. doi: 10.1016/j.gpb.2018.10.002, PMID: 30986482 PMC6522475

