# Supplementary material for: Early flora colonization affects intestinal immunoglobulin G uptake in piglets, which may be mediated by NF-κB-FcRn pathway
Source: Front Microbiol. 2023 Feb 14;14:1136513. doi: 10.3389/fmicb.2023.1136513 (PMC9971964; doi:10.3389/fmicb.2023.1136513)
Supplement: Supplementary file 1 [file Data_Sheet_1.docx]

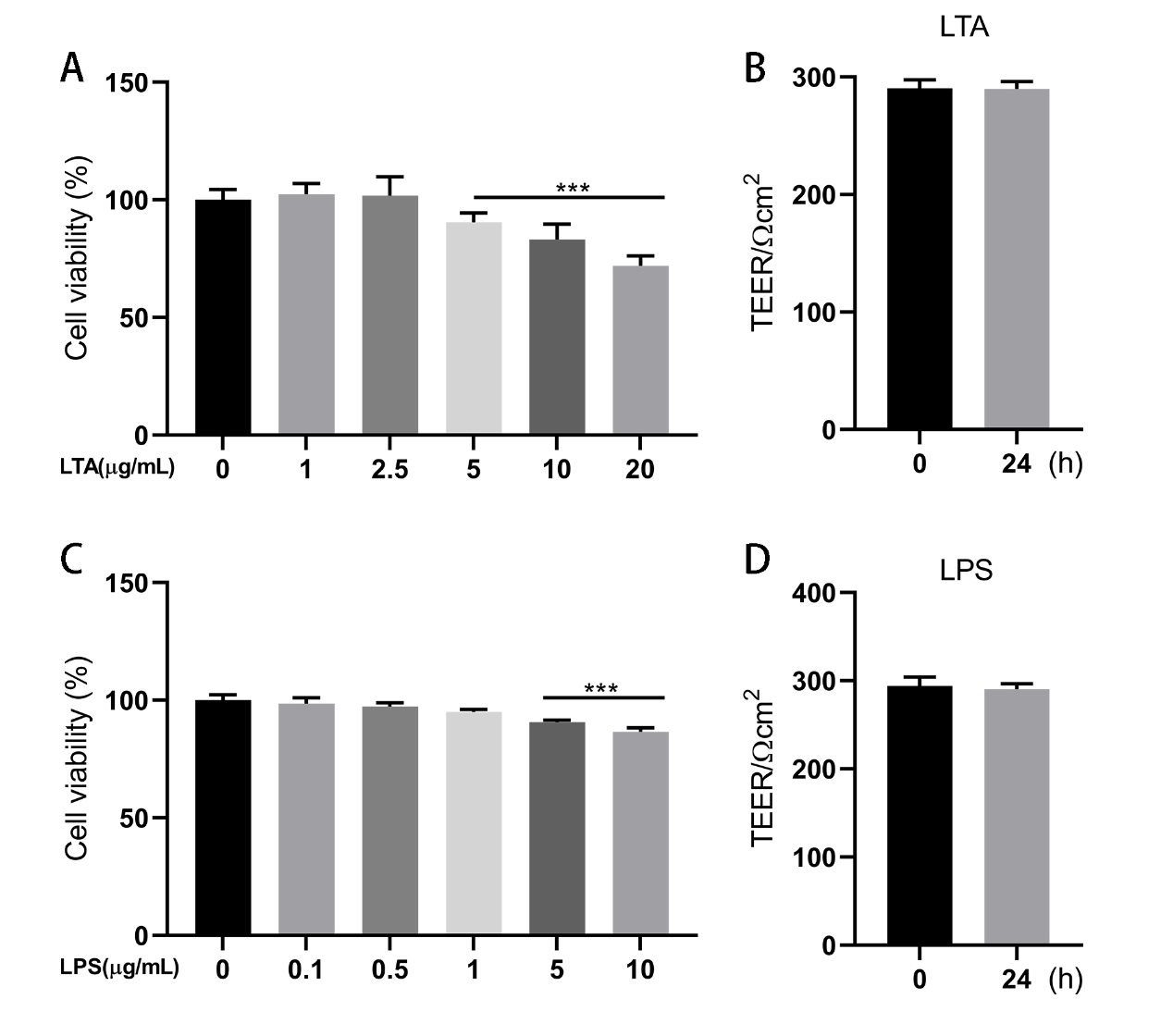


**Fig. S1 Cell viability and trans-epithelial electrical resistance (TEER) of IPEC-J2 cells.**

**(A)** Cell viability of IPEC-J2 cells treated with different concentrations of LTA for 24h. **(B)** The TEER of IPEC-J2 cells after treated with 5 μg/mL LTA for 24 h. **(C)** Cell viability of IPEC-J2 cells treated with different concentrations of LPS for 24h. **(D)** The TEER of IPEC-J2 cells after treated with 1 μg/mL LPS for 24 h.

Data were shown as means ± standard deviations. ^*^*P*<0.05, ^**^*P*<0.01, ^***^*P*<0.001.
